# Biomedical applications of 2D monoelemental materials formed by group VA and VIA: a concise review

**DOI:** 10.1186/s12951-021-00825-4

**Published:** 2021-04-01

**Authors:** Ping Gao, Yufen Xiao, YuliangWang, Leijiao Li, Wenliang Li, Wei Tao

**Affiliations:** 1 School of Chemistry and Environmental Engineering, Changchun University of Science and Technology, Changchun, 130022 China; 2 Center for Nanomedicine and Department of Anesthesiology, Brigham and Women’s Hospital, Harvard Medical School, Boston, MA 02115 USA; 3 Jilin Collaborative Innovation Center for Antibody Engineering, Jilin Medical University, Jilin, 132013 China

**Keywords:** 2D materials, Monoelemental, Group VA and VIA, Biomedical applications

## Abstract

The development of two-dimensional (2D) monoelemental nanomaterials (Xenes) for biomedical applications has generated intensive interest over these years. In this paper, the biomedical applications using Xene-based 2D nanomaterials formed by group VA (e.g., BP, As, Sb, Bi) and VIA (e.g., Se, Te) are elaborated. These 2D Xene-based theranostic nanoplatforms confer some advantages over conventional nanoparticle-based systems, including better photothermal conversion, excellent electrical conductivity, and large surface area. Their versatile and remarkable features allow their implementation for bioimaging and theranostic purposes. This concise review is focused on the current developments in 2D Xenes formed by Group VA and VIA, covering the synthetic methods and various biomedical applications. Lastly, the challenges and future perspectives of 2D Xenes are provided to help us better exploit their excellent performance and use them in practice.

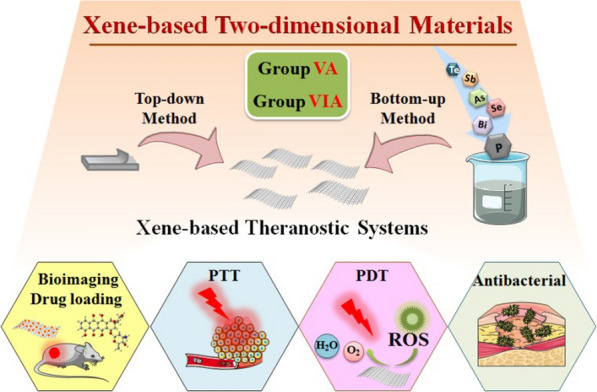

## Background

Two-dimensional (2D) materials are one of the emerging materials, which have more than 100 nm or even several microns with one or a few atomic thicknesses [1]. In 2004, Novoselov and Geim adopted the mechanical separation method to obtain graphene [2]. Graphene is a kind of two-dimensional material with hexagonal honeycomb shape formed by sp^2^ hybridization of carbon protons [3]. Due to its excellent electrical conductivity, high thermal conduct covering sensors, transistors, new energy batteries, hydrogen storage materials, aerospace and so on [4–6]. The great success of graphene has led to a remarkable boom in the development of 2D nanomaterials, such as transition metal dihalide (TMD), nitrides and carbonitrides (MXenes), hexagonal boron nitride (h-BN), graphite phase nitrogen carbide (g-C_3_N_4_), molybdenum disulfide(MoS_2_), layered rare earth hydroxide (LRH), layered double hydride (LDH) and their derivatives [7–10]. 2D nanomaterials with ultrathin lamellate nanostructure exhibit weak interlayer bonding and strong covalent in-plane bonding [11]. There are multifarious unique properties of 2D nanomaterials including the large surface area, the improved chemical and physical reactivities [12–14]. Particularly, the dramatically increased surface area of 2D nanomaterials affects a 2D wave function due to the quantum confinement effects. Consequently, 2D nanomaterials are characterized by impressive photonic [15], catalytic [16], magnetic [17], and electronic properties [18] that differ from those of the bulk counterparts, resulting in a wide-ranging application.

**Fig. 1:**
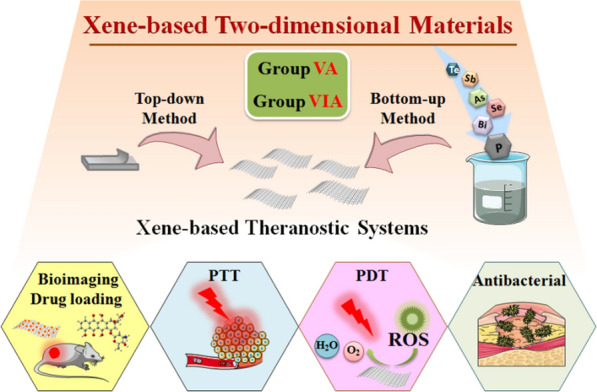
Schematic illustration of the main topics covered in this review

In recent years, 2D monoelemental nanomaterials (Xenes) based on group VA and VIA have gradually entered the researchers' field of vision, which is encouraged by the dramatically development of phosphorene (black phosphorus, BP) [19–22]. Due to the excellent optical and electronic properties, Xenes have been considered to be promising biological theranostic agents to address various challenges in healthcare [23–30]. Xenes can be used as diagnosis agents for computed tomography (CT), photoacoustic imaging (PAI), fluorescence imaging (FI), etc. [31–34]. Moreover, Xenes are employed in disease phototherapy, such as photothermal therapy (PTT) and photodynamic therapy (PDT) against tumor, bacteria and virus [35–41]. The large surface area of Xenes endows them an incomparable high loading capacity of therapeutic and/or fluorescent molecules compared with traditional nanoparticle-based drug delivery platforms. Besides, Xenes also could construct biosensors with the attachment of various biological markers like DNA, etc. [42, 43]. This paper reviews the applications of Xenes formed by Group VA and VIA in biomedical fields. To start with, the synthesis and physical–chemical properties of Xenes are introduced. And then their research progress in biomedicine fields were summarized such as bioimaging, therapy and antibacterial. Finally, on the basis of summarizing the current situation, the paper puts forward the challenges and prospects for future development of Xenes in group VIA and VA (Fig. 1).

## Synthesis of Xenes

The synthetic methods of 2D Xenes can be categorized as top-down fabrication and bottom-up synthesis (Table 1). The top-down method mainly uses mechanical force or molecular intercalation to destroy interlayer bonding, so as to strip the block and obtain a single or multi-layer nanometer sheets [44]. The bottom-up approach is to use chemical conversion methods to directly react different molecular precursors to form nanosheets [45, 46].

**Table 1:** Summary of Xene synthesis method

Periodic Table group	Element	2D form	Morphology	Synthesis methods	Refs
VA	P	Phosphorene	Nanosheets and quantum dots	Mechanical cleavage, ultrasonic exfoliation, electrochemical stripping, plasma-assisted process Other top-down methods, MBE,CVD, Solvent-thermal method	[48–52, 65, 68, 71, 72, 74, 80, 86, 87]
VA	As	Arsenene	Nanosheets and Nanodots	Ultrasonic exfoliation, electrochemical stripping, plasma-assisted process, Other top-down methods	[55, 62, 69, 73]
VA	Sb	Antimonene	Nanosheets and quantum dots	Mechanical cleavage, ultrasonic exfoliation, electrochemical stripping, plasma-assisted process, Other top-down methods, MBE, Vander Waals extension	[26, 50, 56, 67, 70, 73, 75, 89]
VA	Bi	Bismuthene	Nanosheets and quantum dots	Ultrasonic exfoliation, Other top-down methods, MBE, Wet chemistry	[57, 73, 78, 90]
VIA	Se	Selenene	Nanosheets	Ultrasonic exfoliation	[58]
VIA	Te	Tellurene	Nanosheets and quantum dots	Ultrasonic exfoliation, MBE, Solvent-thermal method, thermal evaporation	[59, 79, 88, 91]

### Top-down

The top-down method mainly includes solid-phase stripping and liquid-phase stripping. The solid phase stripping refers to mechanical dissociation stripping. The liquid-phase stripping relies on ultrasonic exfoliation, electrochemical stripping, plasma-assisted process and so on.

#### Mechanical cleavage

**Fig. 2:**
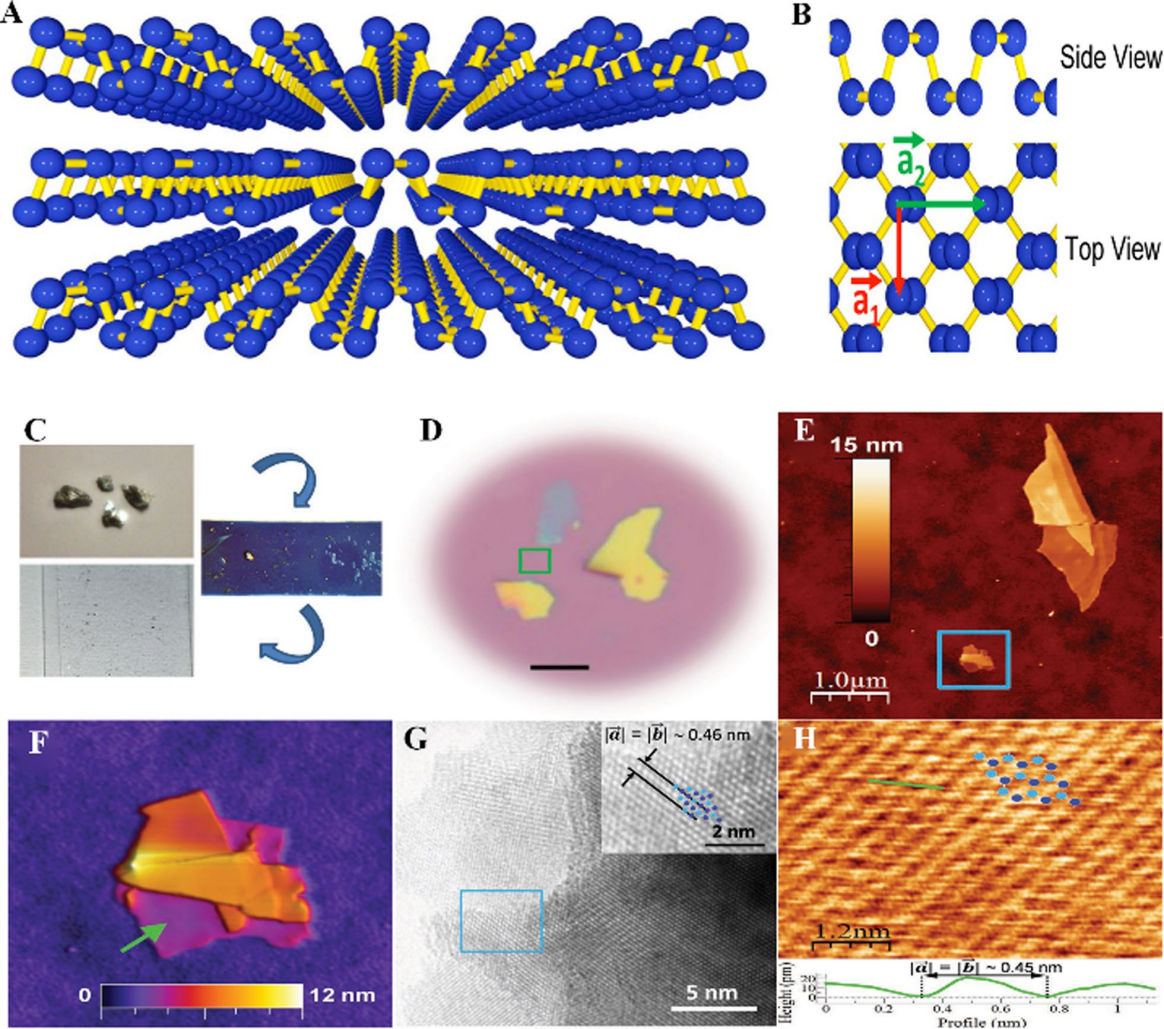
a, **b** Crystal structure of few-layer phosphorene. **a** Perspective side view of few-layer phosphorene. **b** Side and top views of few-layer phosphorene. Reprinted with permission [48], Copyright 2014 American Chemical Society. **c**–**h** Antimonene flakes on SiO2 substrates. **c** Top left, millimeter-size crystals of antimony. Middle right, adhesive tape with sub-millimeter crystals of antimony. Bottom left, polymer on glass slide with micrometer antimony flakes. **d** Optical microscopy image where up to three large flakes of antimony can be seen. **e** AFM topographic image showing two flakes of anti-monene located inside the marked region in **d**. **f** AFM topography of the ≈ 0.2 μm2 antimonene flake inside the blue square in **e** showing terraces of different heights. **g** High-resolution TEM image of a few-layer antimonene flake. The inset is a digital magnification of the area inside the blue rectangle. **h** AFM topography acquired on the bilayer terrace marked with a green arrow in f showing atomic periodicity. Reprinted with permission [50], Copyright 2016 Wiley

Mechanical cleavage is an original and basic approach to strip large layered materials into single or several layers of nanometer sheets by mechanical forces using transparent tape [47]. In recent years, researchers have prepared many 2D nanomaterials by this method. In 2014, BP nanoflakes were firstly obtained by repeatedly peeling the block crystal with transparent tape on Si/SiO_2_ substrate [48, 49]. The BP generated by the method is easy to oxidize in the environment forming irreversible phosphorus oxide compounds. Therefore, it is necessary to use inert gas or vacuum in the preparation process to obtain pure BP nanosheets (Fig. 2a, b). In addition, submillimeter-sized antimonene is prepared directly on the SiO_2_/Si substrate by repeatedly tearing a block crystal over the tape. However, the production yield based on SiO_2_/Si substrate is very low. In 2016, Pablo Ares et al. developed an improved method with the aid of viscoelastic polymer on the surface of SiO_2_ substrate. As shown in Fig. 2c–h, the results of high resolution transmission electron microscopy (TEM) and atomic force microscope (AFM) proved the excellent stability of antimonene prepared by the improved method. And the theoretical calculations of the antimony monolayer also proved the inactive character with water and oxygen [50]. Although mechanical cleavage is an easy and low-cost approach, the yield is relatively low and the controllability and repeatability are dissatisfactory.

#### Ultrasonic exfoliation

**Fig. 3:**
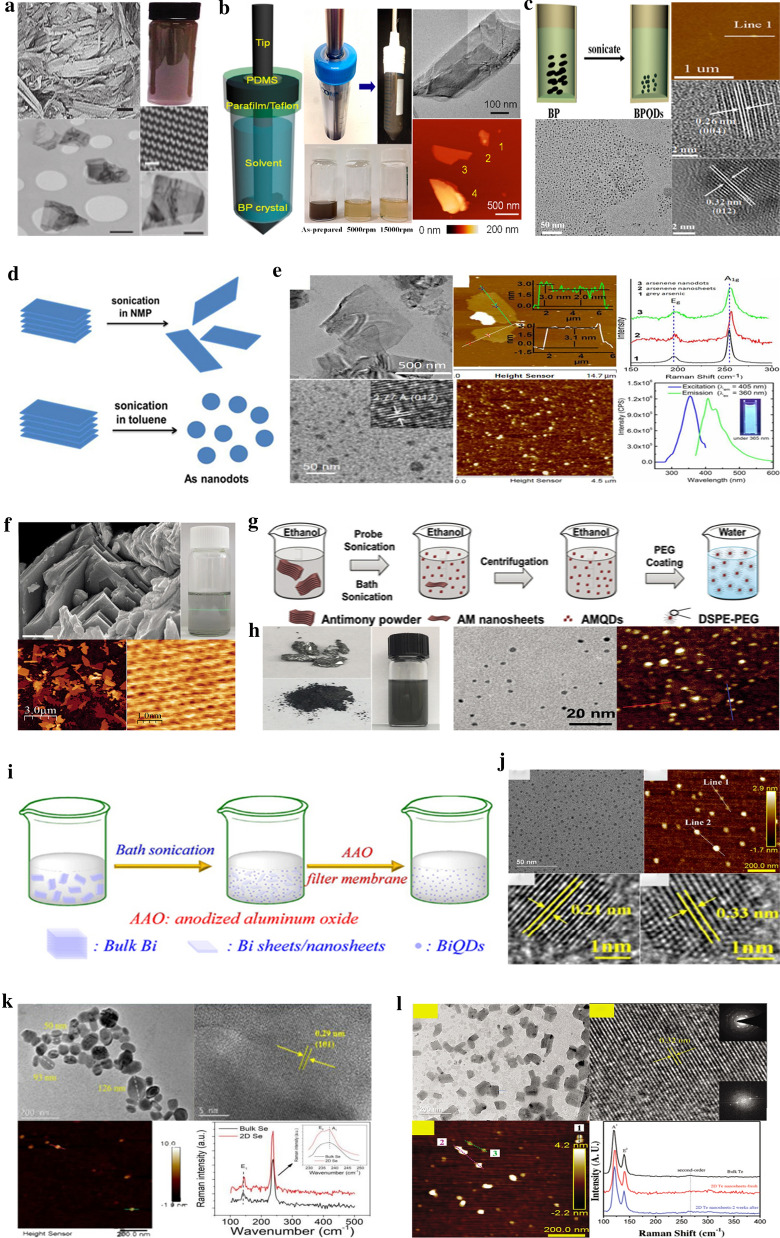
**a** Basic characterization of exfoliated black phosphorous (CHP as solvent, scale bars 100 μm, 500 nm and 1 nm). Reprinted with permission [52], Copyright 2015 Macmillan Publishers. **a** Schematic of solvent exfoliation of BP in NMP solvents via tip ultrasonication and characterization of solvent-exfoliated BP nanosheets. Reprinted with permission [53], Copyright 2015 American Chemical Society. **c** Schematic illustration of the synthesis of BPQDs and experimental morphological (TEM and AFM) images of BPQDs. Reprinted with permission [54], Copyright 2018 Elsevier. **d** Schematic of the preparation of arsenenenanosheets in NMP and nanodots in toluene from grey arsenic. Reprinted with permission [55], Copyright 2018 Royal Society of Chemistry. **e** Experimental morphological (TEM and AFM) images of arsenenenanosheets and nanodots. Reprinted with permission [55], Copyright 2018 Royal Society of Chemistry. **f** Experimental morphological (TEM and AFM) images of antimonene, reprinted with permission [56], Copyright 2016 Wiley. **G **Fabrication of PEG-coated AMQD. Reprinted with permission, [26] Copyright 2017 Wiley. **h** Photos of bulk antimony, antimony powder, AMQDs solution during the preparation, process, TEM and AFM image of AMQDs. Reprinted with permission [26], Copyright 2017 Wiley. **i** Fabrication of BiQDs. Reprinted with permission [57], Copyright 2018 American Chemical Society **j** Experimental morphological (TEM and AFM) images of BiQDs. Reprinted with permission [57], copyright 2018 American Chemical Society. **k** Characterizations of the as-prepared 2D Se through liquid-phase exfoliation. Reprinted with permission [58], Copyright 2019 Elsevier. **l** Characterization of ultrathin 2D Tenanosheets. Reprinted with permission [59], Copyright 2018 Wiley

Recently, ultrasonic assisted liquid phase stripping has attracted dramatically increasing attention. The bulk layered crystal is firstly dispersed in designated medium such as N-cyclohexyl-2-pyrrolidone (CHP), dimethylformamide (DMF), dimethylsulfoxide (DMSO), isopropyl alcohol (IPA), N-methyl-pyrrolidone (NMP) and so on [51]. With the help of ultrasonic wave, abundant bubbles are generated on the surface of crystal through hydroxyl radical mediating or pyrolysis reaction during ultrasonic cavitation and thus ultrathin 2D nanomaterials are produced finally. High-quality, uniform size, low-layer BP nanosheets were prepared with ultrasonic probe by liquid-phase exfoliation in both CHP and NMP solution under cooling condition (Fig. 3a, b) [52, 53]. Furthermore, Song et al. produced BP quantum dots (QDs) by liquid-phase exfoliation with ultrasonic probe in NMP by tuning power and reaction time (Fig. 3c) [54]. These BP QDs possess the average size of 3.5–4.5 nm and the thickness of 1.2–1.6 nm with the lattice fringes of 0.26 nm and 0.32 nm assigned to the (004) and (012) plane of BP. Coincidentally, arsenene (2D arsenic) has also been prepared using gray arsenic as raw material by liquid-phase exfoliation in NMP. As shown in Fig. 3d, e, the thickness of the prepared arsenic flakes is 6–12 atomic layers and the size is 100–350 nm in average. The Raman spectra showed thickness dependence. Nonetheless, the products are only arsenic nanoparticles by replacing solvent medium with toluene when other conditions are held constant [55]. The lamellar β-antimonene was obtained by ultrasonication in a mixture of IPA and water (IPA:water = 4:1) at 400 W for 40 min (Fig. 3f). The transverse size is greater than 1-3μm^2^ accompanied with the thicknesses 4 nm [56]. Tao et al. synthesized the ultra-small and uniform size antimony quantum dots by the two-step combined ultrasonic strategy of ultrasound probe sonication and ice bath ultrasound in ethanol, and modified them with 1,2-Distearoyl-sn-glycero-3-phosphoethanolamine-N-[methoxy (poly ethylene glycol)] (DSPE-mPEG) to improve their dispersion and stability in physiological media. As presented in Fig. 3g, h, the average size of the exposed antimony quantum dots is approximately 2.8 nm, and the average thickness is approximately 1.6 nm. The average size of antimony quantum dots modified by mPEG is 3.9 nm and the average thickness is 2.6 nm [26].

Ultrathin bismuthene QDs can be produced by liquid-phase exfoliation in NMP for 48 h under the power of 400 W (Fig. 3i) [57]. Selenene (2D selenium) has been experimentally obtained with 200 W power liquid-phase exfoliation along with 360 W ultrasound treatment subsequently. As shown in Fig. 3k, the obtained selenium nanosheets with the size of 20–130 nm and the thickness of less than 10 nm have been successfully applied in photoelectric fields [58]. Tellurene (2D Tellurium) could also be obtained by liquid-phase exfoliation in IPA after pulverizing. The prepared 2D Te nanosheets with good stability keep the crystallization characteristics during the stripping process (Fig. 3l) [59]. For size/thickness-controllable preparation of 2D Xenes by ultrasound-assisted liquid phase exfoliation, the decisive factor should be the power and the reaction time of ultrasonic. And the first critical factor should be the appropriate selection of solvent. The above mentioned organic solvent molecules could be embedded into the interlayer of layered materials like wedges, which is beneficial to break the weak van der Waals forces and gain ultrathin nano-lamina. The decisive factor should be the power and the reaction time of ultrasonicto size/thickness-controllable preparation of 2D Xenes [60–62]. Ultrasonic exfoliation is one of the most effective methods for preparing 2D Xenes, and it is also considered as a universal method for various 2D Xenes.

#### Electrochemical stripping

**Fig. 4:**
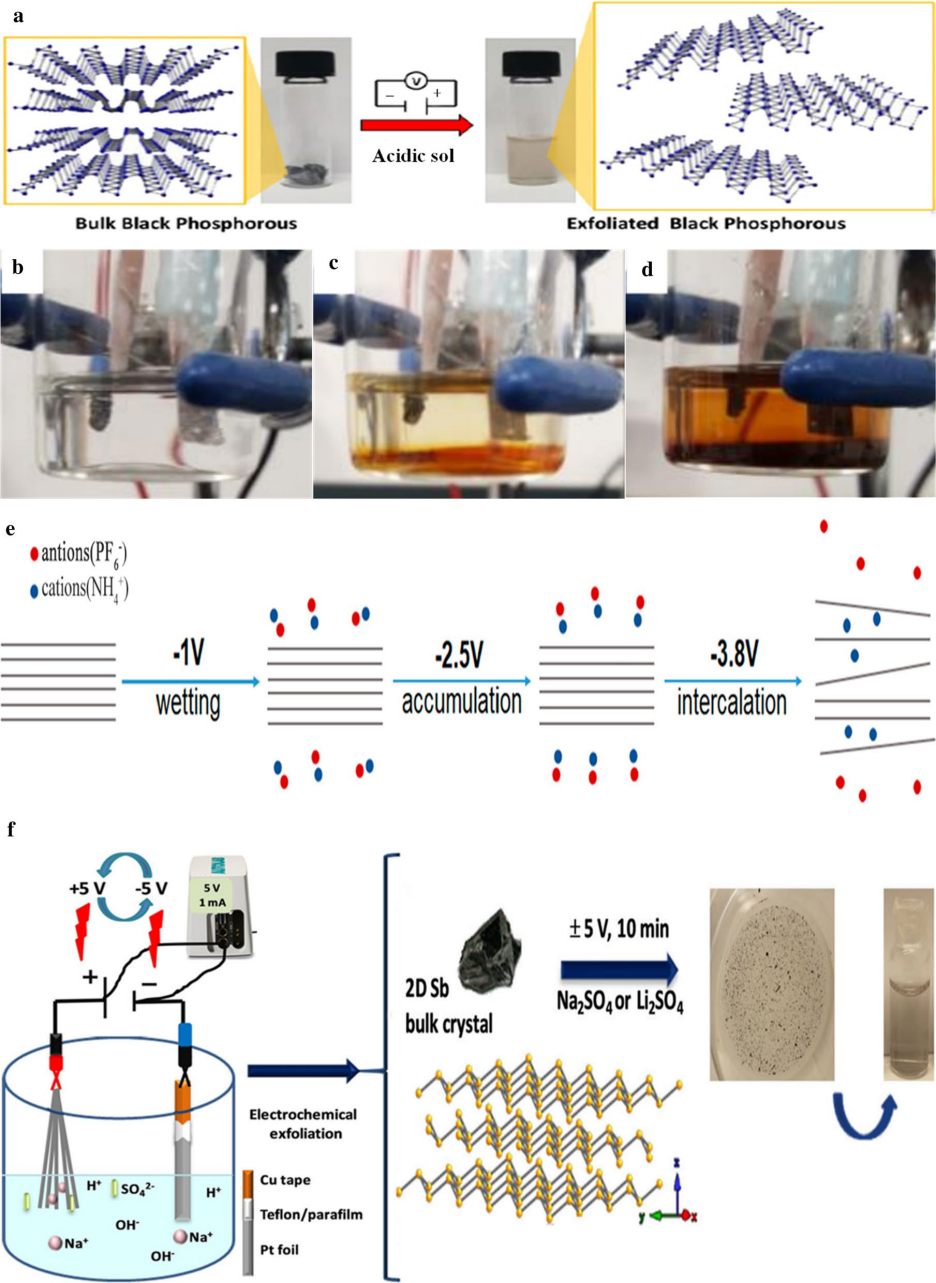
**a** Schematic of the black phosphorus exfoliation procedure. Snapshot of the electrochemical setup with BP flake anode and Pt foil cathode separated in acidic solution (0.5 M H_2_SO_4_) by a fixed distance of 2 cm at **b** no potential applied, **c** after 20 min applying a voltage of + 3 V and **d** after 2 h process. A-D Reprinted with permission [65], Copyright 2017 Wiley. **e** Low-Potential Electrochemical Exfoliation. Scheme. Low-potential electrochemical exfoliation of native As toward (mono)few-layer arsenene: (blue dots) cations (NH4+); (red dots) anions (PF6−). Reprinted with permission [66], Copyright 2017 Royal Society of Chemistry. **f** General scheme for the electrochemical exfoliation of layered Sb crystals into 2D sheets. Reprinted with permission [67], Copyright 2020 Wiley

Electrochemical stripping including ion intercalation method and anodizing method has been used in the synthesis of graphene. Recently, 2D Xenes were also obtained with the assistance of mild ultrasound by ion intercalation method. The embedding cations (such as K^+^, Na^+^, Li^+^, etc.) could enlarge the space and weaken the van der Waals force between layers. Besides, these cations could also react with water to generate hydrogen, which is beneficial to the separation between layers as well and thus improves the productivity [63, 64]. For instance, BP nanosheets were successfully prepared from black phosphorus film by ion intercalation method. As illustrated in Fig. 4a–d, Adriano Ambrosi et al. used black phosphorus film as anode, platinum plate as cathode, and connected with copper strip and put it into H_2_SO_4_ solution at given voltage. The solution gradually turned yellow/orange and finally turned deep orange. After cleaning and vacuum drying, the small particles at the bottom were re-dispersed in dimethylformamide (DMF). The green/gray dispersion was obtained by ultrasonic treatment, and BP nanosheets were obtained after vacuum drying at 40 ℃ [65].

The preparation of arsenene (2D arsenic) was achieved by electrochemical stripping. Bulk arsenic was used as cathode (working electrode), platinum and Ag/AgNO_3_ were used as anode and reference electrode, respectively. In this reaction, ammonium hexafluorophosphate (NH_4_PF_4_) was selected as electrolyte and then accumulated on the surface of anode (Fig. 4e). The multilayered arsenic pieces further screen out 2D arsenene with thickness of 0.6 nm. This electrochemical assisted method will facilitate the application of arsenene in a new generation of electronic devices [66]. Using platinum and antimony as electrodes in Na_2_SO_4_ and Li_2_SO_4_ electrolytes at 5 V for 2 h to obtain antimonene (2D antimony) [67]. The voltage polarity and the type of electrolyte have an important influence on the peeling production (Fig. 4f). Electrochemical stripping is a scalable method to obtain biocompatible Xenes. Compared with the traditional method, this method can prepare high yield and low cost 2D materials with high requirements, which have been realized in phosphorene, MoS_2_ and other materials.

#### Plasma assisted process

This method is important for the fabrication of high-performance phosphenyl nanoelectronic devices. Phosphorene could be prepared by pyrolysis of black phosphorus crystal on SiO_2_/Si substrate using Ar^+^ plasma (13.56 MHz RF source), 30 pair pressure and 30 W power at room temperature. According to the Raman spectra, the frequency of A_2g_ mode becomes hard with the decrease of atomic thickness, while the frequencies of β_2g_ and A_1g_ modules are almost constant. This may be due to the anisotropic structure changes of different thickness of phosphoranes [68].

**Fig. 5:**
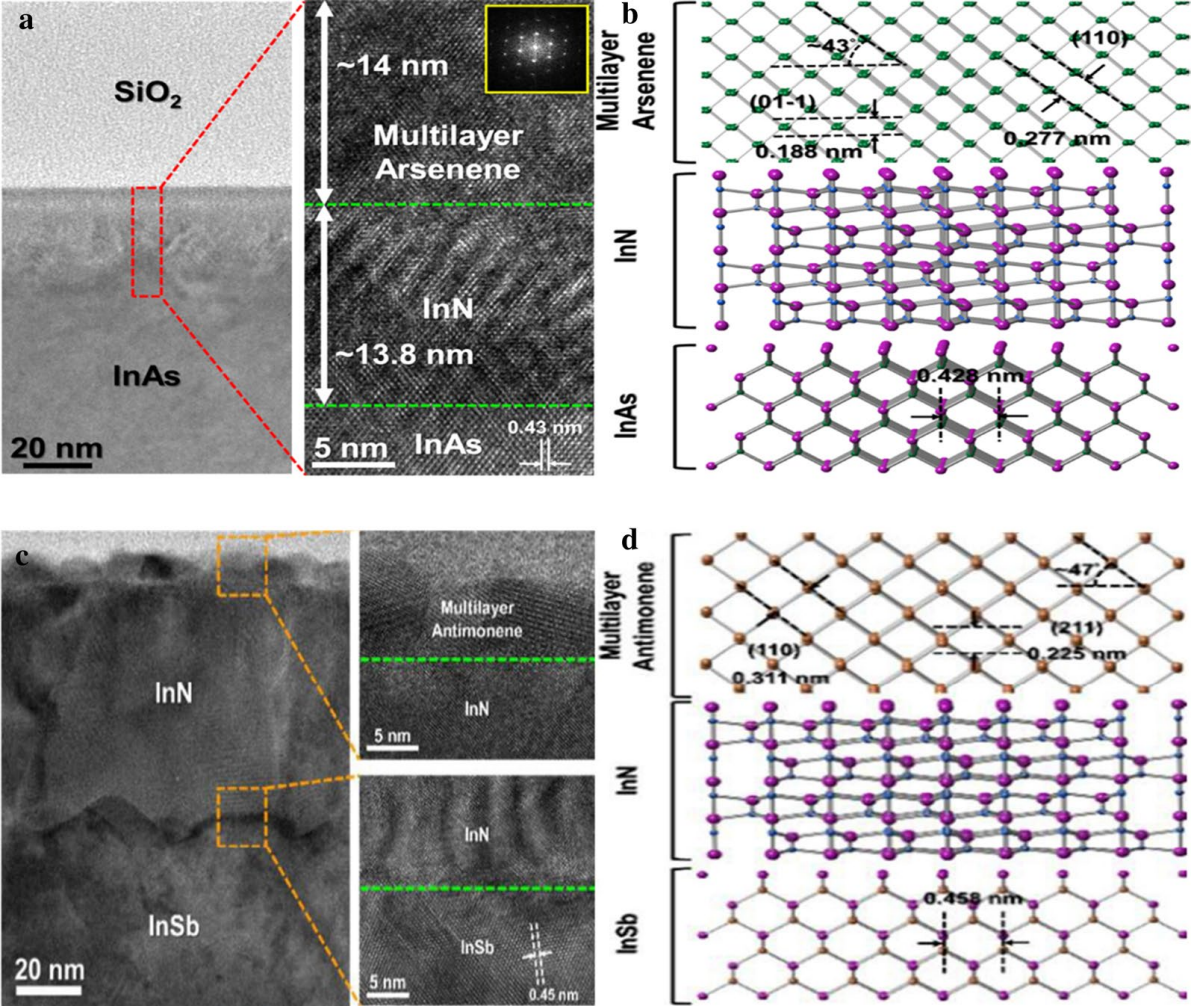
**a** TEM image of the multilayer arsenene/InN/InAs. (Inset:diffraction pattern of multilayer arsenene). **b** Thetheoretical atomic model of multilayer arsenene/InN/InAs layer structure. Reprinted with permission [69], Copyright 2016 American Chemical Society. **c** TEM image of the multilayer antimonene/InN/InSb. **d** The theoretical atomic model of multilayer antimonene/InN/InSb layer structure.Reprinted with permission [70], Copyright 2016 Royal Society of Chemistry

Moreover, Tsai et al. synthesized multilayer arsenic nanosheets using plasma-assisted processes on the InAs substrate (Fig. 5a, b) [69]. There are many factors that affect the size, thickness and morphology of arsenic nanosheets including annealing time and the plasma exposure time. The greater the plasma power, the higher the arsenic phonon mode strength and the more crystal defects. The heterostructure includes multilayered arsenic, InN and InAs substrates, respectively. The multilayer antimony nanoribbons can be prepared by immersing the InSb substrate in a 50–200 W N_2_ plasma for 30–60 min at 10^−1^ torr and then drying and annealing in N_2_/H_2_ (10/1, V/V) atmosphere for 30–60 min (Fig. 5c, d) [70]. However, this method is suitable for mass production of 2D nanomaterials due to its high cost and high equipment requirements.

#### Other top-down methods

In addition to the above-mentioned preparation methods of 2D Xenes, there are many other preparation methods. Xu et al. mixed large black phosphorus crystals with NMP in a 1:1 ratio (weight ratio w/w) under vigorous stirring by a household mixer for 40 min at 250 W power to produce a single layer or several layers of BP nanosheets [71]. A simple and efficient microwave-assisted liquid-phase stripping method was performed to prepare BP nanosheets [72]. A large amount of black phosphorus was dissolved in a small amount of NMP and heated in a 600 W microwave system at 50 °C for 20–40 min. And then the intermediate products were transferred to a 220 W microwave system at 70 ℃ for 3 min to obtain a dispersion of stable BP nanosheets. In addition, by using hepatocholic acid as surfactant, the As, Sb and Bi bulk crystals were dispersed and stirred acutely in the kitchen mixer for 2 h to obtain 2D As, Sb and Bi nanosheets [73]. TEM images showed that arsenic formed the largest thin section with folded structure due to its high anisotropy, and the layered structure was small. The thicknesses of the synthetic 2D antimony was 10 times less than that of 2D arsenic. The electronic transfer ability, sensing performance and electrochemical performance of these materials were measured.

### Bottom-up

The bottom-up method can be divided into physical preparation, chemical preparation and other bottom-up methods. Physical preparation includes molecular beam epitaxy (MBE) in physical vapor deposition, and chemical preparation includes chemical vapor deposition (CVD), hydrothermal method and solvothermal method.

#### Molecular beam epitaxial

**Fig. 6:**
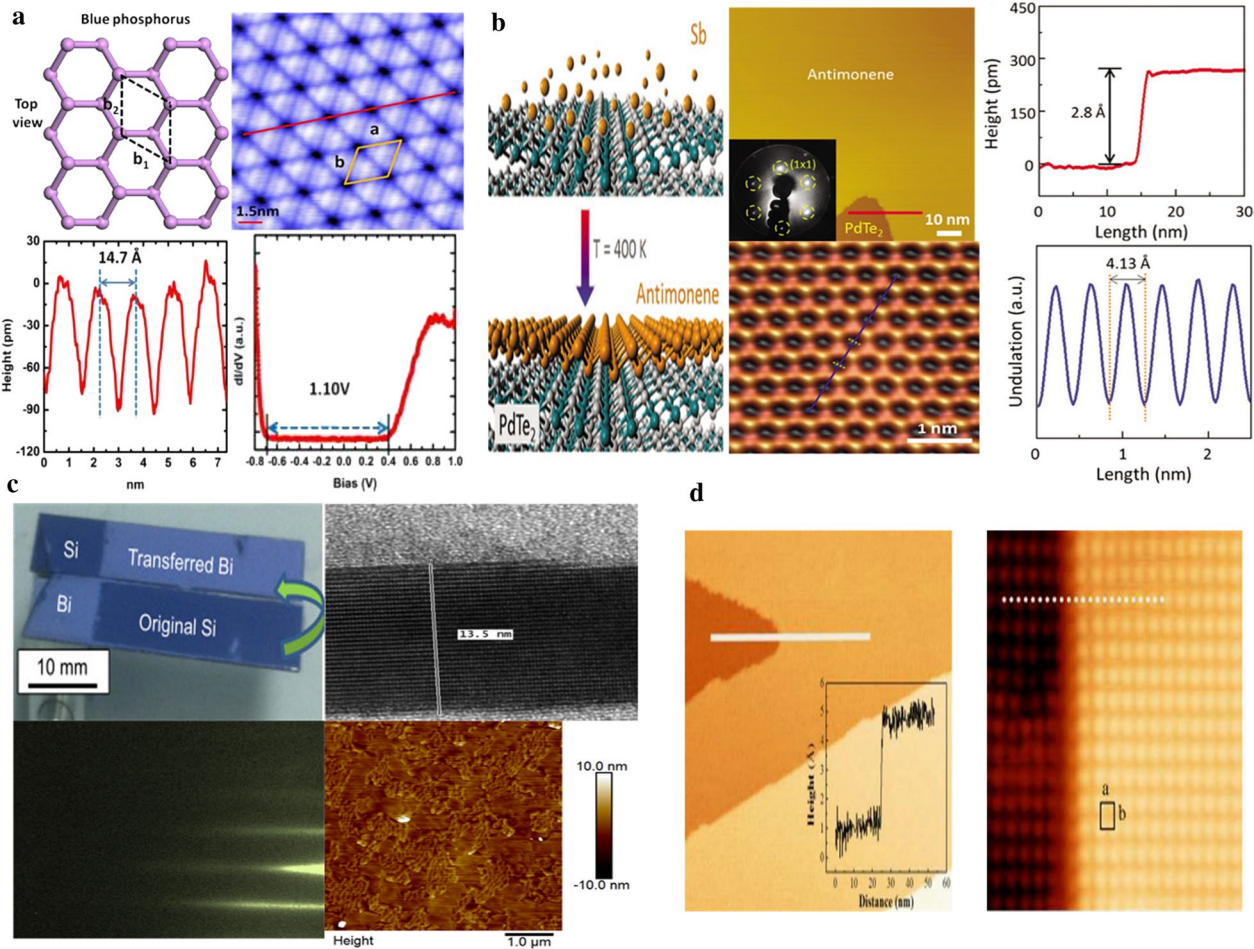
**a** Atomic model of blue phosphorus and experimental morphological (STM) images of phosphorus. Reprinted with permission [74], Copyright 2016 American Chemical Society. **b** Monolayer antimonene formed on PdTe2 substrate, experimental morphological (TEM and AFM) images of antimonene. Reprinted with permission [75], Copyright 2017 Wiley. **c** Morphological of the transferred Bi films. Reprinted with permission [78], Copyright 2016 American Chemical Society. **d** Left: Topographic image (size: 100 × 100 nm2, sample bias: 1 V) of an epitaxial Te film showing an atomically flat terraces separated by steps of height of ~ 4 Å. (The inset presents a line profile taken along the white line drawn in the image). Right: Atomic resolution STM image (size 8 × 8 nm2, bias: 0.6 V) showing rectangular lattices as highlighted by the black rectangle. Reprinted with permission [79], Copyright 2017 Royal Society of Chemistry

MBE is a vacuum coating technology in the synthesis of modern semiconductor device materials. The thickness, crystal orientation and doping amount of Xenes can be accurately controlled by this method. For instance, using black phosphorus as the precursor, P_4_ molecules were condensed from the vapor phase on the surface of Au (111) substrate. After deposition and annealing, monolayer phosphorus with regular hexagonal morphology was formed [74]. As shown in Fig. 6a, high resolution scanning tunnelling microscopy (HRSTM) showed that the single phosphorus layer was highly ordered, and each dark center was surrounded by six triangles. The average distance between dark centers was about 14.7 Å. The measured electronic band gap of a single layer of P_4_ was about 1.10 eV, which indicated that it was a new kind of two-dimensional semiconductor material. It was consistent with the theoretical predictions reported by Zhu et al. The DFT calculations predicted the new form of 2D phosphorus with a flat "zigzag ridge", which was similar to that of silene with layered honeycomb structure. 2D antimony was also obtained by MBE method besides 2D phosphorus. Monolayer antimonene with regular orientation and order array could grow on a chemical stable layered TMD substrate (PdTe_2_) [75]. The small mismatch (less than 2.3%) of the surface lattice constant between the substrate (PdTe_2_, 4.01 Å) and that of free-standing antimonene (4.01 Å) should be a critical factor for the formation of the 2D antimony film. The resultant antimony membrane with honeycomb lattices is similar to graphene is highly ordered and symmetric. The height of antimony coating was 2.8 Å, which was close to the calculated height of monolayer of antimony (3.38 Å) (Fig. 6b).

The large band gap of 2D monolayer antimony indicated the potential applications for electronic and photoelectric devices. Silicon is identified as a suitable alternative substrate to prepare Xenes by MBE method [76, 77]. For example, full-wafer monocrystalline bismuth films with the thickness within the range of 4 ~ 50 nm have been grown on Si (111) substrates by MBE (Fig. 6c) [78]. Si (111) substrate was firstly soaked with dilute hydrofluoric acid to remove the natural oxide and then the substrate was loaded into a high vacuum and baked at high temperature within 20 min to prevent secondary oxidation. Bismuthene grows at a rate of 0.2 Å/s at room temperature observed by TEM. Bismuthene possesses good 2D topological structure due to its quantum size effect, low carrier density and spin coupling effect. As for Group VIA, tellurene (2D tellurium) was recently grown on both graphene/6H-SiC (001) and high orientation pyrolysis graphene (HOPG) using MBE technology [79]. As shown in Fig. 6d, the lattice of tellurene is rectangular and the surface lattice constant is consistent with the theoretical lattice constant. The tellurium thin films feature semiconductor characters, and the band gap decreases with the increase of thickness. MBE method could control the growth of Xenes at atomic scale due to the slow deposition rate, however, the difficult operation limits their more applications.

#### Chemical Vapor Deposition (CVD)

CVD technology has gradually become an important route for synthetic of uniform 2D inorganic materials. BP thin films with an average area of above 3 μm^2^ were prepared from red phosphorus successfully with in situ chemical vapor deposition method [80]. The film was composed of four phosphorus atomic layers. In recent years, many new 2D nanomaterials have been prepared by CVD technology. The controllable growth of Sb_2_Te_3_ nanosheets occurs on the surface of SiO_2_ [81]. Bismuth oxide monocrystalline films could be prepared on amorphous substrates by aerogel-assisted CVD [82]. Chang et al. used CVD technology to prepare continuous InSe films possessing high stability against oxidation [83]. CVD technology has many advantages in the preparation of two-dimensional materials. The deposition rate can be controlled by adjusting the process parameters, such as gas pressure, gas velocity, temperature rising rate and heat preservation time. CVD technology is simple and relatively low in cost, which is suitable for large-scale production and wide applications [84, 85].

#### Solvent-thermal method

**Figure7:**
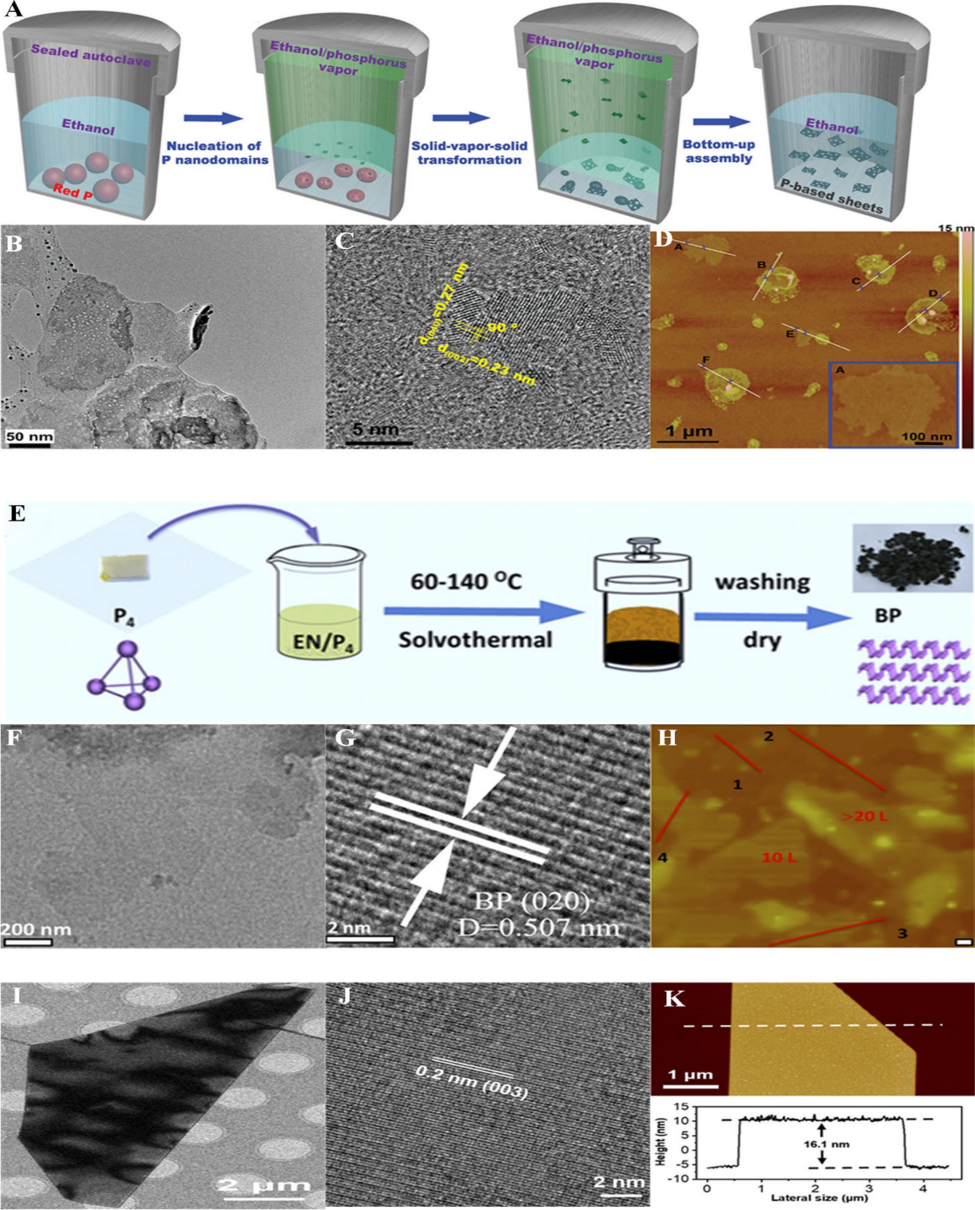
**a** The morphology evolution of the bulk red phosphorus materials during the high-temperature solvothermal reaction. **b** Low-magnification of TEM images of the holey phosphorus-based nanosheets. Reprinted with permission [86], Copyright 2016 Wiley. **c** HRTEM image of phosphorus-based nanosheets, showing the amorphous regions with some polycrystalline structure. **d** AFM image. **e** Synthetic protocol of the black phosphorus. **f** TEM image of the BP nanosheets. **g** HRTEM image corresponding to F. Lattice Image of a BP flake shows d spacing of the (020) plane of orthorhombic BP. **h** AFM image. Reprinted with permission [87], Copyright 2018 PNAS. **i** TEM images of the tellurium nanoflakes. **j** Corresponding HR-TEM image. **k** AFM image of typical tellurium nanoflake (top) and the corresponding height profile (bottom), scale bar is 1 µm. Reprinted with permission [88], Copyright 2018 American Chemical Society

Solvent-thermal method is a common method to synthesize nanomaterials. An impressive body of literature indicates that solvent-thermal approach is a reliable method for the synthesis of BP nanosheets. For instance, high yield of BP nanosheets (30%) was prepared at 400 ℃ (Fig. 7a) [86]. After vacuum drying, the uniform 2D nanocrystals with the transverse size of 1 μm and the thickness of 0.5–4 nm were obtained, which was characterized by less than 8 layers of black phosphorus atoms (Fig. 7b–d). The lattice fringes of 0.27 nm and 0.23 nm were on the (040) and (002) planes of orthogonal BP. The resultant BP nanosheets feature excellent electrochemical properties as the positive electrode of lithium ion battery. Tian et al. [87] took white phosphorus as raw material and ethylenediamine as solvent to obtain BP nanosheets with a few atomic layers through solvent-thermal approach (Fig. 7e–h). More interestingly, the higher the solvothermal reaction temperature (in 60–140 ℃ range), the higher the yield of BP production. The thickness of the synthetic BP nanosheets was within the scope of 1–15 nm, which was comprised of 2–28 atomic layers. It was conducive to the expansion of BP application scope in the field of optoelectronic devices.

As for tellurium, sodium tellurite (Na_2_TeO_3_) is a common tellurium source with hydrazine hydrate (N_2_H_4_•H_2_O) as the reducing agent for the preparation of 2D tellurium by solvent-thermal approach [88]. As shown in Fig. 7i–k, the measurements of TEM and AFM consistently demonstrated that the uniform dimensions (about 15 μm) with the thickness of 10–30 nm of tellurene can be achieved by this process. The result indicated that Te nanosheets were continuous lattices with a lattice constant of 2 Å corresponding to the (003) plane of Te. Moreover, the thickness of 2D tellurium nanocrystals could be adjusted from single molecule membrane to 10 nm by controlling reaction conditions. The resultant 2D tellurium nanosheets have high stability. In conclusion, the yield and morphology of 2D nanocrystals are dramatically influenced by reaction temperature, raw materials, solvents, and surfactants in this process.

#### Other bottom-up methods

In order to obtain 2D materials with high crystallinity by CVD technology, the perfect match in symmetry and lattice constants between the epitaxial layer and substrate is a key index. Van der Waals epitaxy technology is recently proposed to seed the epitaxial growth of 2D materials on the substrate without suspended bond to avoid the above restrictions. Ten atomic layered antimonene was obtained by van der Waals epitaxy from antimony polyhedrons on a bare (001) plane fluorochemical substrate (KMg_3_(AlSi_3_O_10_)F_2_). The antimony vapor in the heat was transported downstream with Ar/H_2_ mixed gas resulting in thin antimonene films with less than 4 nm [89]. Wet chemistry is another common bottom-up method to prepare Xenes. Bismuthene was synthesized by this method. The black sediment was obtained from the mixture of Bi(NO)_3_·5H_2_O, added water, ethylene glycol and hydrazine hydrate (volume ratio 6:3:1) under vigorous stirring it at 80 ℃ for 8 h [90]. More recently, Zhao et al. [91] deposited ultrathin tellurium films by thermal evaporation at low temperature (− 80 ℃) when the pressure reached 2 × 10^–6^ mbar. Tellurium thin film prepared by this method has high Hall mobility and is a kind of high performance p-type field effect transistors (FET) materials with good switching characteristics. It has a broad application prospect in electronic devices and monolithic 3D circuits. Tan et al. also reported the use of low temperature (− 110 °C) thermal evaporation to prepare Se_x_Te_1-x_ thin films with continuous adjustable band gap from 0.31 eV (Te) to 1.87 eV (Se) [92]. The films have great application potential in the manufacture of low cost, high performance, high resolution short-wave infrared (SWIR) photodetectors and imaging sensors.

## Application of Xenes in biomedicine

Therapeutic applications of 2D Xenes have witnessed rapid growth for biomedical applications in recent years. Because of their outstanding physical, chemical, electronic and optical properties, 2D Xenes have been explored in a variety of biomedical applications, such as bioimaging, photothermal therapy (PTT), photodynamic therapy (PDT), chemotherapy and antibacterial. These various adopted strategies in biomedical applications are described as follows.

### Bioimaging

Multimode imaging is paramount importance for the identification and diagnosis of the disease. It is a routine technique to visualize morphological details in cells and tissues to avoid unnecessary biopsies and reduce patient suffering [93]. In this section, we will discuss the applications of 2D Xenes in fluorescence imaging (FI), photoacoustic imaging (PAI), and X-ray computed tomography (CT) imaging.

**Fig. 8:**
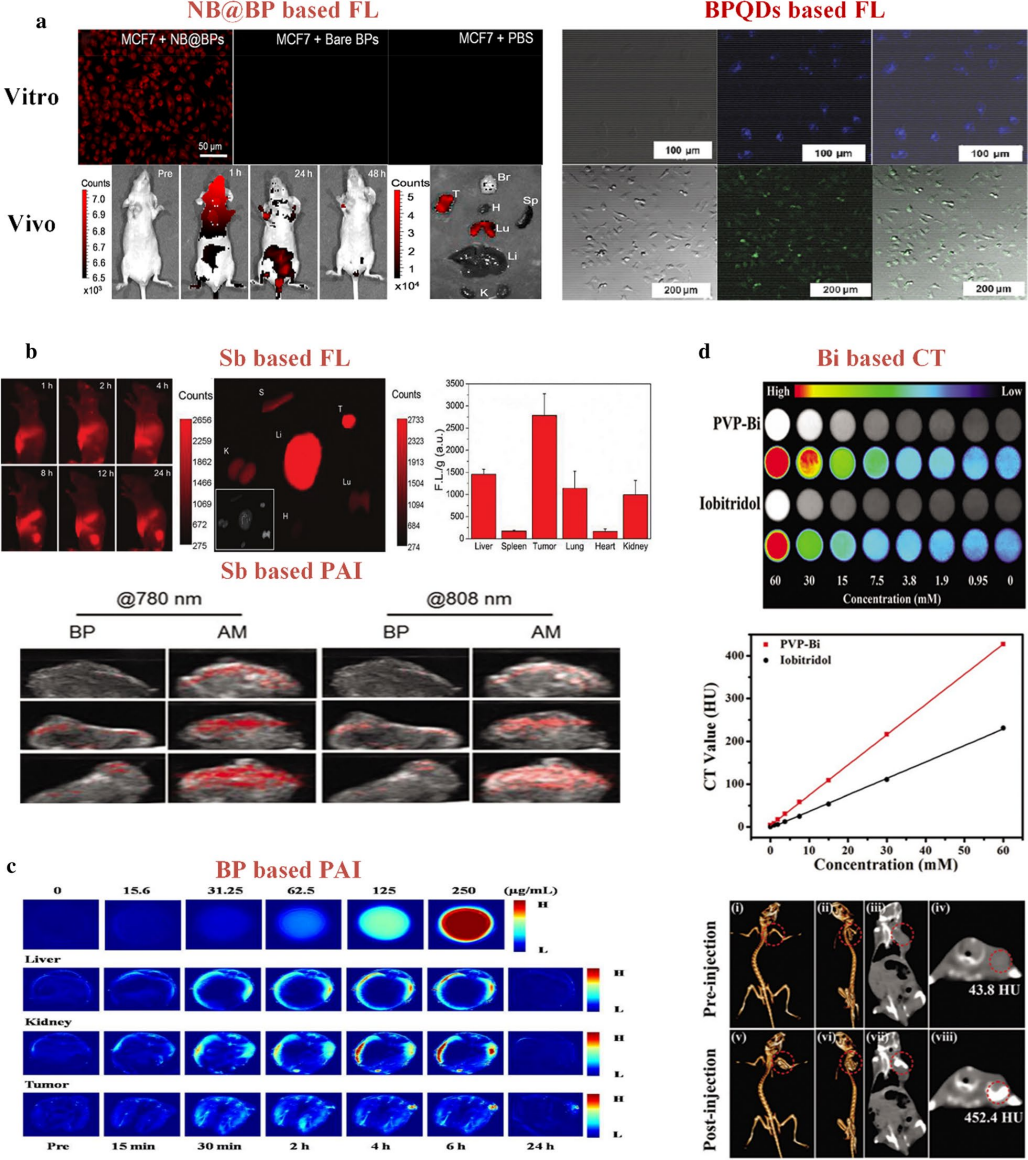
Emerging Xene-based bioimaging. **a** NB@BP-based fluorescence imaging (FL) and BP quantum dots based fluorescence imaging (FL). Reprinted with permission [94], Copyright 2017 American Chemical Society. Reprinted with permission [96], Copyright 2016 Wiley. **b** Antimonene-based fluorescence imaging (FL) and photoacoustic imaging (PAI). Reprinted with permission [27], Copyright2018 Wiley. **c**) BP-based photoacoustic imaging (PAI). Reprinted with permission [97], Copyright 2016 Elsevier. **d** Bismuthene-based X-ray computed tomography (CT) imaging. Reprinted with permission [101], Copyright 2017 Wiley

FI is the visualization of fluorescent dyes or proteins as labels for molecular processes or structures. FI is a common biological imaging model to achieve observations including the location and dynamics of gene or protein expression and molecular interactions in cells and tissues. 2D Xenes with ultra-high specific surface area can efficiently load fluorescent dyes for fluorescence imaging. BP has been widely demonstrated as an excellent fluorescent dye delivery platform to achieve the requirement of real-time imaging for living tissue. For example, the modified nile blue dye (NB) was covalently modified BP nanosheets to form NB@BP, which could label MCF-7 tumor sites with red fluorescence and achieve effective tumor ablation under near-infrared irradiation (Fig. 8a) [94]. Fluoresce in isothiocyanate (FITC)-labelled PEG-BP NSs has been demonstrated that it could enter HeLa cells through cavity-dependent endocytosis and macroendocytosis [95]. This result provides an available evidence for deep investigating the transport mechanism and distribution of BP in cells. PEGylated BP loading Cy7 could achieve a good accumulation in the tumor sites model resulting in vivo imaging [21]. BP quantum dots have good biocompatibility, low toxicity, spontaneous degradation analogous to BP nanosheets, especially intrinsic fluorescence properties making it a broad application prospect in bioimaging. Lee et al. found that black phosphor quantum dots emitted blue fluorescence in HeLa cells under UV irradiation and green fluorescence under visible light irradiation (Fig. 8a) [96]. Additionally, antimonene has also been explored in fluorescence imaging field showing good accumulation and retention in the mouse model of breast cancer cell inoculation (Fig. 8b) [27]. Cy5.5-labeled PEGylated antimonene nanosheets were absorbed into MCF-7 cells by mass endocytosis and cave-independent endocytosis, and then transported by early endocyt-late endocyt-lysosome pathway.

PAI, which is based on the photoacoustic effect, is an emerging diagnostic modality to achieve the detection of laser-irradiated tissue-induced pressure waves. PAI has been applied to the imaging of cancer, wound healing, disorders in the brain, and gene expression, among others. Due to the thickness-dependent quantum size effect and adjustable crystal structure of Xenes, it is an extremely attractive PAI reagent to achieve the sectional or three-dimensional images of tissues and organs. For example, PEGylated BP nanosheets have been demonstrated as a good PAI agent [97]. As shown in the Fig. 8c, PAI signal was enhanced with the increase of concentration of PEGylated BP nanosheets. PA signals in tumor sites were strong for several hours following the injection of PEGylated BP nanosheets, indicating the low tissue-attenuation coefficient and the prominent accumulation in tumor focus in comparison with the other organs. In addition to BP, Tao et al. have developed the uniform antimonene nanosheets and successfully applied it as PAI agents in bioimaging [27]. They reported that the PAI signal of antimonene nanosheets was stronger than that of BP nanosheets, which was more suitable for in vitro and in vivo imaging. Recently, other forms of Xenes including ultrasmall bismuth [98] (2D Bi) nanoparticles and tellurene [99] (2D Te) nanosheets were also reported to be ideal candidate for the application in PAI.

Combining with special computer-aided X-ray technology, CT utilizes ionizing radiation to provide 3D images of biological tissues and fine cross-sections with high resolution and deep penetration noninvasively. Given the advantages of long half-life, easy modification and specific enrichment in tumor region, Xene-based nanomaterials also have great potential in the application of CT imaging. Bismuth possesses good X-ray attenuation performance, which is relatively cheaper and lower toxic compared with the conventional CT agents. The edge value of bismuth (K = 90.5 keV) is three times greater than that of iodine-based medicine (K = 33.2 keV), which is a common clinic CT agent. Therefore, bismuth-based nanomaterials could maximize the X-ray absorption efficiency and have good CT imaging potential [100]. Lei et al. synthesized ultra-small PVP-coated Bi nanodots and demonstrated the improved CT imaging results [101]. The CT imaging resolution is triple as high as that of the traditional iodine-based contrast agent (Fig. 8d). Liu et al. prepared Bi@Gel (BGPS) and proved that CT signal was enhanced with the increase of BGPS concentration [102]. The Hounsfield unit slope of BGPS is 6.464 Humm^−1^, higher than that of iohexol (4.28 HumM^−1^) resulting in excellent CT imaging. The obvious CT signals could be detected at the tumor site after injection for 24 h, which further proved that the CT imaging performance of BGPS effectively accumulated in the lesion. In this context, Xene-based nanomaterials are widely considered as a potential alternative in vitro and in vivo imaging. Furthermore, combining these diverse imaging methods could improve the spatial and temporary sensitivity of imaging systems for tumor therapy.

### Therapeutic applications

The treatment of the disease, especially targeted therapy, is essential inclinical medicine [103–106]. Chemotherapy, photothermal therapy (PTT) and photodynamic therapy (PDT) are the most common therapeutic methods based on Xene nanostructures.

**Fig. 9:**
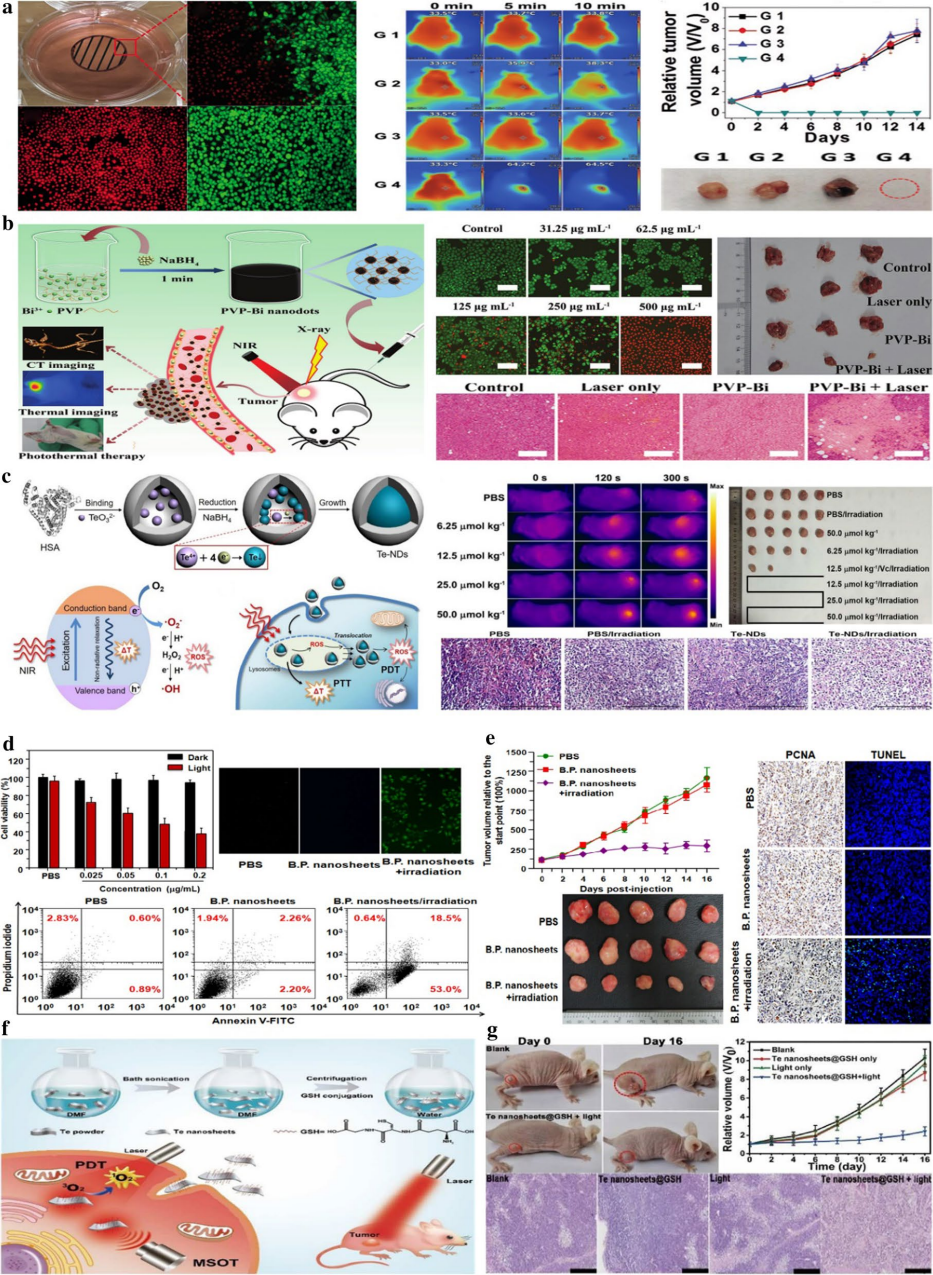
Emerging Xene-based therapeutic applications. **a** Antimonene-based photothermal therapy (PTT). Reprinted with permission [26], Copyright 2017 Wiley. **b** Bismuth-based photothermal therapy (PTT). Reprinted with permission [101], Copyright 2017 Wiley. **c **Tellurium Nanodots-based photothermal therapy (PTT) and Photodynamic therapy (PDT) Reprinted with permission [22], Copyright 2017 American Chemical Society. **d**, **e** Black phosphorous-based Photodynamic therapy (PDT). Reprinted with permission [107], Copyright 2015American Chemical Society. **f**, **g** Tellurene-based photodynamic therapy (PDT). Reprinted with permission [109], Copyright 2018 Royal Society of Chemistry

PTT is a physical treatment approach to destruct cancer using local hyperthermia generated by photothermal agent with high photothermal conversion efficiency. Xenes with strong absorption in the region of near-infrared light, has been widely used as photothermal agents for PTT due to their unique optical performance [22]. For example, the fatality rate of BP-PEG nanosheets under 1 W/cm^2^ laser irradiation on HeLa cells reached 90%, indicating that BP-PEG nanosheets can promote the death of cancer cells by good photothermal effect. In vivo experiments showed that BP-PEG could enhance the anti-tumor effect through the combination of PTT and chemotherapy, and no tissue damage was found in the main organs. Additionally, the photothermal conversion efficiency of Ce6-modified BP nanosheets were reported as high as 43.6%. Apart from BP in group VA, 2D antimony quantum dots (AM QDs) have been prepared and functionalized by PEG. The temperature of PEG-coated AMQDs could increase up to 50 ℃ at the concentration of 200 μg/mL, indicating the extremely high photothermal conversion rate (45.5%). More importantly, PEG-coated AM QDs could degrade rapidly under the action of near-infrared light after executing PTT against tumor. And in vivo experiment against MCF-7 indicates that 2D AM QDs have the best antitumor effect without recurrence and obvious side effects induced by local hyperthermia (Fig. 9a) [26]. It was reported that PVP encapsulated Bi QDs (PVP-Bi QDs) also exhibited good photothermal conversion capability under laser irradiation of 808 nm at the power of 1.3 w/cm^2^. The temperature of PVP-Bi QDs sharply rose up to 49.5℃ within a short time, indicating PVP-Bi QDs could rapidly convert near-infrared light into heat energy. The measured photothermal conversion efficiency was about 30%. After treating U14 tumor with PVP-Bi quantum dots combined with laser irradiation, the cytotoxicity and effects on normal cells were negligible (Fig. 9b) [102]. As for Group VIA, Te-based nanomaterials have been proposed to perform PTT to achieve the purpose of tumor ablation. Yang and co-workers presented the synthesis of bifunctional tellurium nanodots, which could not only achieve effective photothermal transformation but also generate ROS to mediate apoptosis in tumor cells (Fig. 9c) [107].

PDT is nowadays another form of phototherapy to destruct cancer, which possesses the therapeutic procedure exerting a selective cytotoxic activity toward malignant cells. In the presence of oxygen, the photosensitizer or photosensitizing drug could produce reactive oxygen species (ROS) by irradiation at a certain wavelength corresponding to an absorbance band of the sensitizer. The irreversible oxidative damage to the cancer cells in malignant tissues is induced by the generated ROS and there is the minimal normal tissue toxicity. Clinical studies have shown that PDT can be curative and significantly improve the reduction of recurrence risk, particularly in early stage tumors. Various advantages make it a valuable therapeutic schedule for combination treatments.

Fortunately, Xene-based photosensitizers are expected to overcome the forementioned limitations. Xene itself is not only an ideal candidate for PDT but also can be loaded with photosensitizing drug due to its super high specific surface area. Wang et al. reported that BP nanosheets produced singlet oxygen under 660 nm laser irradiation at the power of 1 W/cm^2^ to induce apoptosis of tumor cells, and the apoptosis rate was 71.5% [108]. As shown in Fig. 9d, e, both in vitro and in vivo experimental results indicated that the BP nanosheets have great potential for using as a photosensitizer. Numerous studies show that the synergistic effect of PTT and PDT can effectively inhibit tumor growth. Yang et al. constructed a dual functional Ce6-modified BP nanosheets to realize PDT/PTT synergistic treatment [109]. This combination was endowed with good biocompatibility and obvious synergistic effect. It was also less-invasive for normal cells, heart, liver, spleen, kidney and other major organs. Lin et al. prepared glutathione coated tellurium nanosheets (Te@GSH), which showed a noteworthy PDT capability with a high quantum yield about 0.91 of singlet oxygen (^1^O_2_) under 670 nm light irradiation (Fig. 9f, g) [98]. Te@GSH could produce ^1^O_2_ and effectively inhibit the growth of tumor cells. Hematoxylin–eosin (H&E) staining images showed that the tumor sections in the Te@GSH light group were seriously damaged, while the morphology of other cells was normal and the physiological morphology of the viscera was not changed. Te@GSH was further proved a safe and reliable PDT agent. In addition to the two-dimensional Xenes materials mentioned above, the applications of BP QDs and tellurium nanoparticles in PDT have also been reported.

**Fig. 10:**
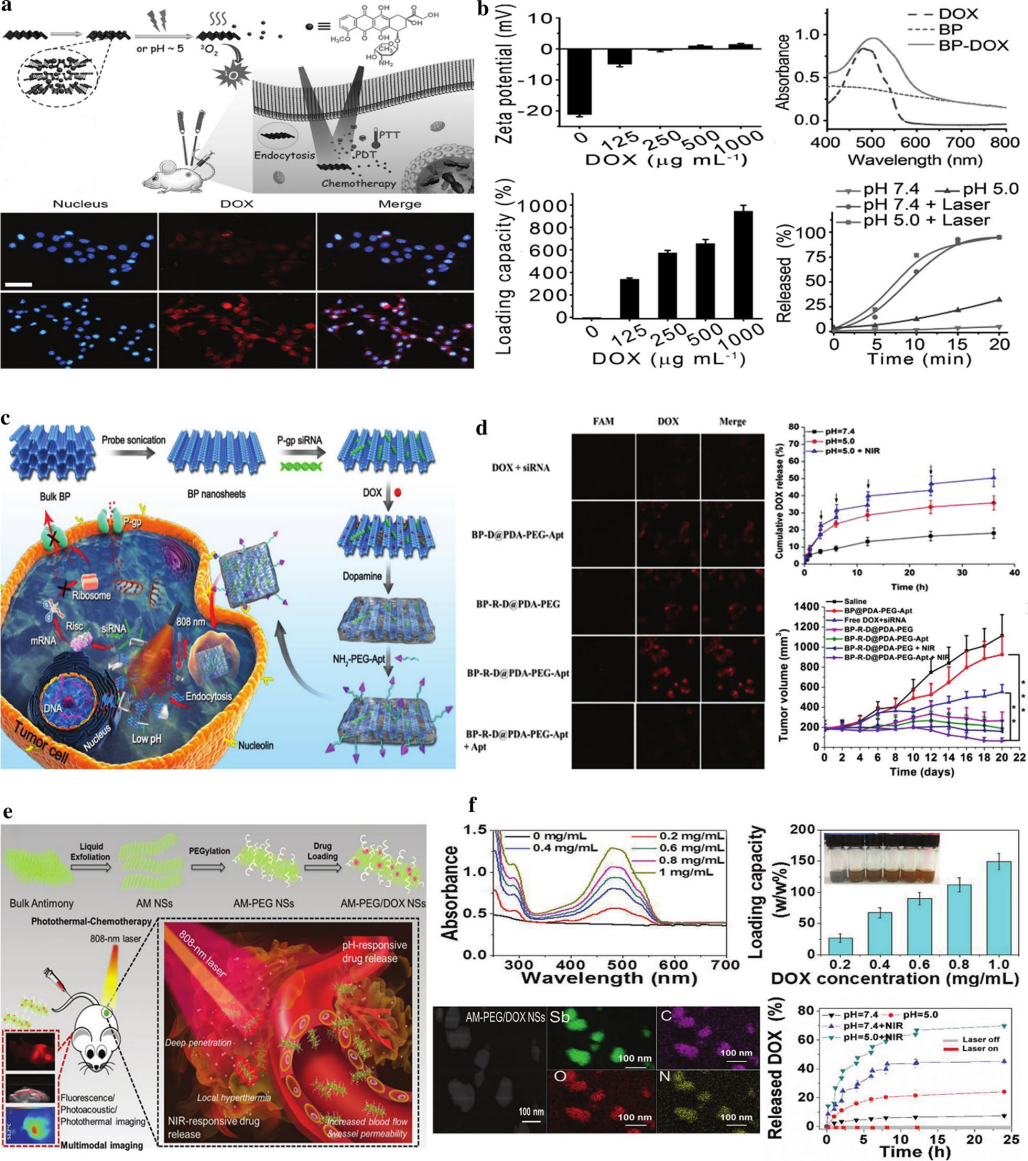
**a**, **b** Phosphorene-based drug delivery systems. Reprinted with permission [28], Copyright 2017 Wiley. **c**, **d** Polydopamine-modified black phosphorous-based drug delivery systems Reprinted with permission [112], Copyright 2019 Wiley. **e**, **f** Antimonene-based drug delivery systems. Reprinted with permission [27], Copyright 2018 Wiley

Although there are some drawbacks, chemotherapy is still the main method for cancer therapy in clinical present. The satisfying chemotherapy effect could be achieved via combining 2D Xenes with anticancer functional drug. 2D Xenes have unique lamellar structure, adjustable atomic layer thickness, large specific surface area and easy surface functionalization. These advantages provide an important basis for high drug loading and effective drug delivery [28, 110]. As a newly developed 2D material, BP nanosheets are metal-free layered semiconductors with variable band gap, high surface reactivity, strong biodegradability, large specific surface area, which can efficiently load drug molecules, antibodies and biological molecules. Therefore, combining anticarcinogen with BP nanosheets is an effective approach for chemotherapy. For example, Chen and co-workers presented the synthesis of a multifunctional system, which combined BP nanosheets and lipophilic drugs (doxorubicin, DOX) through electrostatic effect (Fig. 10a, b) [111]. The loading capacity of DOX reached up to 950%, which was much higher than the drug loading of conventional deliveries reported previously. More importantly, the release rate of the drug at pH 5 was 6 times higher than that at pH 7, demonstrating that the drug was beneficial to release at a lower pH and the release profile was pH-dependent. Because of the acidic tumor microenvironment, BP-DOX is favorable for drug release within the tumor. BP has photothermal effect, which can further promote the release of DOX. Under the irradiation of 808 nm laser, the release rate of DOX was up to 90%. In addition to DOX, chloroquine (CQ) has also been successfully loaded onto BP nanosheets [112]. The BP nanosheets loaded with CQ enter the cell lysosomes, realizing the synergistic treatment of photothermal therapy and chemotherapy, which significantly improved the therapeutic effect of cancer. Following these studies, Zeng group employed polydopamine (PDA) to innovate a facile and low-cost surface modification strategy to endow BP nanosheets with good water-solubility and bio-compatibility (Fig. 10c, d) [113]. DOX and P-gp siRNA physically adsorbed on the surface of PDA coated BP-based drug delivery for multidrug resistant cancer treatment. The release rate of DOX and P-gp siRNA could be effectively controlled by adjusting pH and near infrared laser irradiation. In vivo experiments showed that the system could successfully introduce drugs into tumor cells, and exhibited a significant synergistic therapeutic effect on multidrug resistant breast cancer. In a word, by constructing a PDA-modified BP nanosheets multifunctional drug delivery platform, the water-solubility, bio-compatibility and photothermal performance could be improved. It could not only achieve selective cell targeting, but also show effective inhibition of tumor cell proliferation through multi-mode combination therapy.

The multifunctional drug delivery system provides a new direction for targeted chemotherapy and gene therapy for multidrug resistant cancers. Tao et al. has reported the coating of antimony nanosheets (2D Sb) multifunctional system with amphiphilic PEG polymer for targeted delivery of chemotherapy drugs and photothermal therapy [27]. As illustrated in Fig. 10e, f, antimony nanosheets were encapsulated by PEG grafted amphiphilic polymer to offer a "hydrophobic arm" with the feasibility of physically absorbance "catching" lipophilic drugs (DOX) by hydrophobic interaction. The obtained drug loading capacity was about 150% (weight ratio) and the measured drug release rate was 69.8% at pH 5 under the near-infrared irradiation. AM-PEG/DOX has deep photoinduced penetration and excellent photothermal conversion capability. The apoptosis rate of MCF-7 cells was about 91.5% and the tumor growth inhibition rate (TGI) of this strategy reached 98.1%, which was significantly higher than that of monotherapy. It was the first time to report the photon drug delivery platform of 2D Sb nanosheets, which may indicate a new starting point in cancer treatment research. Apparently, Xenes as drug delivery have attracted extensive attention and made rapid progress in research. Although there are still huge challenges, it is believed that in the near future, Xenes will make great breakthroughs and realize clinical applications.

### Antibacterial

**Fig. 11:**
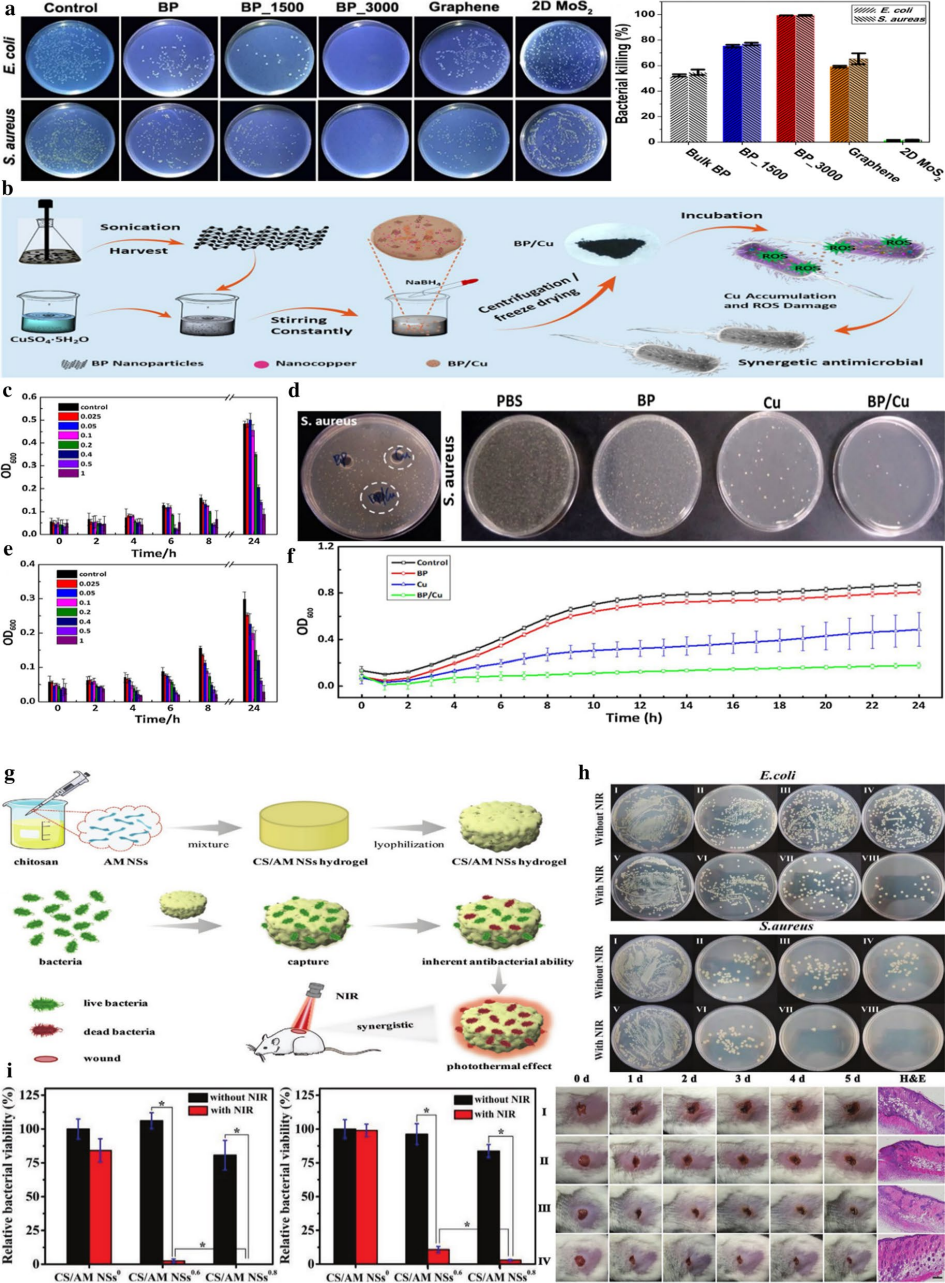
Emerging Xene-based antimicrobial application. **a** Phosphorene-based antimicrobial application. Reprinted with permission [115], Copyright 2018 Royal Society of Chemistry. **b**–**f** BP loaded copper (BP/Cu)-based antimicrobial application. Reprinted with Reprinted with permission [116]. Copyright 2020 Elsevier. **g** Schematic illustration of the preparation of CS/AM NSs hydrogel and its use in treating bacterial wound infection. **h**, **i** Pictures of antibacterial effect in vitro and photographic images and tissue sections of wounds treated by Staphylococcus aureus infection. Reprinted with permission [117], Copyright 2020 Wiley

Bacterial infection is a process in which bacteria invade and cause local or systemic damage to the host, thus showing its pathogenicity and inducing the occurrence of disease. Currently, the most common treatment for bacterial infections is antibiotic therapy. However, due to the emergence of multi-drug resistant superbugs, traditional antibiotics are no longer able to solve the arisen problem of bacterial infection. An overuse of antibiotics has further accelerated the creation of antibiotic-resistant germs [114, 115]. To date, tremendous efforts have been devoted to settle the awful bacterial drug resistance. Xenes have good membrane permeability, excellent biocompatibility, large specific surface area and easy surface modification, so they can interact with bacterial membranes better and improve antibacterial effect distinctly. At present, great progress has been made in the development of Xenes based antibacterial agents [29, 30]. For instance, Sun et al. employed BP nanosheets as an antibacterial agent to against gram-negative escherichia coli (E. coli) and gram-positive staphylococcus aureus (S. aureas) by means of 808 nm irradiation [116]. The sterilization rate was as high as 99.2%, far higher than that of graphene and 2D MoS_2_, showing significant antibacterial ability (Fig. 11a). The effect of BP nanosheets on staphylococcus aureus were slightly larger than that of E. coli, which may be related to the different microstructure of different cell wall. Within a certain concentration range, the bacterial lethality improved and the cytotoxicity would be negligible with the increasing of BP concentration, indicating the good biocompatibility and the great potential in antibiotic area. Ouyang' group constructed a novel Ag@BP nanostructure on the substrate of BP nanosheets [117]. Under near-infrared light, BP substrate has excellent photothermal effect and can rapidly destroy bacterial membrane. Synergistically Ag^+^ was released slowly by oxidative dissolution mechanism to inhibit the proliferation of bacteria. They demonstrated that higher amounts of methicillin-resistant staphylococcus aureus (MRSA) death (93%) caused by Ag@BP than BP alone. After treatment with Ag@BP, the skin tissue was intact and the inflammation was effectively inhibited. The antibacterial effect of Ag@BP is mediated by local high temperature and oxidative stress, and has nothing to do with the structure of bacteria, thus avoiding the occurrence of drug-resistant bacteria. Ag@BP have good biocompatibility and biosafety making them have great potential in clinical application in the future. This work also laid a foundation for the treatment of drug-resistant bacteria based on two-dimensional semiconductor and other antibacterial material microspheres. Zhang et al. directly synthesized a new type of copper-carrying (BP/Cu) nanocomposite by one-step reduction method, and studied its antibacterial mechanism (Fig. 11b–f) [118]. Active lone pair electrons could allodially transfer from BP to the surface of metal according to the in vivo experiments. The interaction between BP and copper leads to the increase of ROS production mainly including hydroxyl radicals and superoxide anion. The generated ROS active species could directly damage the cell membrane, phospholipid or membrane protein, thereby destroying the bacterial structure and further inducing bacterial death.

In addition to BP, antimony nanosheets have great potential for synergistic antibacterial applications. Liu et al. introduced antimony nanosheets into the center of chitosan (CS) network to construct a composite hydrogel (CS/AM-NSs hydrogel) with excellent antibacterial properties (Fig. 11g–i) [119]. The interaction between CS and bacterial membrane made the bacteria accumulate on the surface of the composite hydrogel. The bactericidal property of the composite could kill most bacteria, and the photothermal properties of the antimony nanosheets could eliminate the residual bacteria. The antibacterial test results in vitro showed that the killing rate of CS/AM-NSS0.8 hydrogel against E. coli and S. aureus was 97.1% and 100%, respectively. The results showed that the synergistic effect of CS, AM-NSs and PTT could effectively kill bacteria and further promote wound healing. There was no obvious inflammation, injury or necrosis, no aggregation of materials in major tissues and organs, and the toxicity was relatively weak in vivo. The material is expected to be widely used in bandages to treat bacterial wound infections. 2D antimony based nanomaterials was used in antimicrobial therapy for the first time, providing a future direction of biomedical applications for 2D Xenes. Rangrazi et al. synthesized chitosan groomed selenium nanoparticles (CTS-Se NPs) directly by a simple chemical reduction method with ascorbic acid as reducing agent [120]. The results showed that CTS-Se NPs exhibited obvious bactericidal effect on gram-positive bacteria, streptococcus sanguis, staphylococcus aureus and enterococcus faecalis even at extremely low concentration of CTS-Se NPs. Moreover, this system had no obvious inhibitory effect on gram-positive pseudomonas aeruginosa and salmonella typhimurium, which indicated that it possessed selective antibacterial function. CTS-Se NPs have great reference and application value in medical field such as forehead sterilization of medical devices and mouthwash for dental diseases. In brief, 2D Xenes are conducive to contact with bacteria and penetrate the cell membrane due to their internal features containing high specific surface area, light-induced ROS production, outstanding photothermal conversion efficiency.

## Conclusion and prospect

In this review, we summarized the recent developments in 2D Xenes (group VA and group VIA) in terms of design, synthesis and biomedical applications. Numerous typical examples were enumerated to demonstrate the various aspects of 2D Xenes in detail. Admittedly, the research of 2D Xenes has made substantial advances in recent years, however, several technical challenges remain, which impose barriers to their practical applications. On one hand, controllable preparation of 2D Xenes is of primary importance, including morphology, composition and adjustable surface properties. There is no standard method to synthesize 2D Xenes and how to prepare 2D Xenes on a large scale is still a huge challenge. Therefore, more profound understanding still needs to be explored in future. On another hand, an urgent task is to pinpoint the biosecurity of 2D Xenes including the accumulation, the accurate retention time in the lesion and the actual clearance mechanism. 2D Xenes are ingested, the prognosis within the body and the generated effect on nervous and immune system still needs to be further systematic studied. Therefore, further nanotoxicology and pharmacokinetics studies on 2D Xene-based theranostic platform should be carefully confirmed.

In conclusion, 2D Xenes are facing unprecedented challenges and opportunities in biomedical fields. The realization of theoretical knowledge and clinical applications of 2D Xenes nanomaterials requires the joint efforts of all researchers. It is believed that with the continuous development of nanotechnology, 2D Xenes will be applied in a variety of biomedical fields in the near future.

## Data Availability

Not applicable.

## Abbreviations

2D: Two-dimensional
Xenes: Monoelemental nanomaterials
TMD: Transition metal dihalide
h-BN: Hexagonal boron nitride
g-C_3_N_4_: Graphite phase nitrogen carbide
MoS_2_: Molybdenum disulfide
LRH: Layered rare earth hydroxide
LDH: Layered double hydride
CT: Computed tomography
PAI: Photoacoustic imaging
FI: Fluorescence imaging
PTT: Photothermal therapy
PDT: Photodynamic therapy
TEM: Transmission electron microscopy
AFM: Atomic force microscope
CHP: N-cyclohexyl-2-pyrrolidone
DMF: Dimethylformamide
DMSO: Dimethylsulfoxide
IPA: Isopropyl alcohol
NMP: N-methyl-pyrrolidone
QDs: Quantum dots
DSPE-mPEG: 1,2-Distearoyl-sn-glycero-3-phosphoethanolamine-*N*-[methoxy (poly ethylene glycol)]
NH_4_PF_4_: Ammonium hexafluorophosphate
MBE: Molecular beam epitaxy
CVD: Chemical vapor deposition
HRSTM: High resolution scanning tunneling microscopy
Na_2_TeO_3_: Sodium tellurite
N_2_H_4_·H_2_O: Hydrazine hydrate
NB: Nile blue
FITC: Fluoresce in isothiocyanate
ROS: Reactive oxygen species
Te@GSH: Glutathione coated tellurium nanosheets
H&E: Hematoxylin–eosin
TGI: Tumor growth inhibition rate
*E. coli*: *Escherichia coli*
*S. aureas*: *Staphylococcus aureus*
MRSA: Methicillin-resistant staphylococcus aureus
CS: Chitosan
